# Conservation measures or hotspots of disease transmission? Agri-environment schemes can reduce disease prevalence in pollinator communities

**DOI:** 10.1098/rstb.2022.0004

**Published:** 2023-03-27

**Authors:** Robyn Manley, Vincent Doublet, Owen N. Wright, Toby Doyle, Isobel Refoy, Sophie Hedges, David Pascall, Claire Carvell, Mark J. F. Brown, Lena Wilfert

**Affiliations:** ^1^ Department of Biosciences, University of Exeter, Streatham Campus, Exeter EX4 4QD, UK; ^2^ Institute of Evolutionary Ecology and Conservation Genomics, University of Ulm, 89069 Ulm, Germany; ^3^ Centre for Ecology and Conservation, University of Exeter, Penryn TR10 9FE, UK; ^4^ Department of Psychology, University of Exeter, Streatham Campus, Exeter EX4 4QG, UK; ^5^ MRC Biostatistics Unit, University of Cambridge, Cambridge CB2 0SR, UK; ^6^ UK Centre for Ecology & Hydrology, Benson Lane, Crowmarsh Gifford, Wallingford OX10 8BB, UK; ^7^ Centre for Ecology, Evolution, and Behaviour, Department of Biological Sciences, Royal Holloway University of London, Egham TW20 0EX, UK

**Keywords:** pollinators, conservation, disease ecology, agri-environment schemes, bees, dilution

## Abstract

Insects are under pressure from agricultural intensification. To protect pollinators, conservation measures such as the EU agri-environment schemes (AES) promote planting wildflowers along fields. However, this can potentially alter disease ecology by serving as transmission hubs or by diluting infections. We tested this by measuring plant–pollinator interactions and virus infections (DWV-A, DWV-B and ABPV) across pollinator communities in agricultural landscapes over a year. AES had a direct effect on DWV-B, reducing prevalence and load in honeybees, with a tentative general dilution effect on load in early summer. DWV-A prevalence was reduced both under AES and with increasing niche overlap between competent hosts, likely via a dilution effect. By contrast, AES had no impact on ABPV, its prevalence driven by the proportion of bumblebees in the community. Epidemiological differences were also reflected in the virus phylogenies, with DWV-B showing recent rapid expansion, while DWV-A and ABPV showed slower growth rates and geographical population structure. Phylogenies indicate that all three viruses freely circulate across their host populations. Our study illustrates how complex interactions between environmental, ecological and evolutionary factors may influence wildlife disease dynamics. Supporting pollinator nutrition can mitigate the transmission of important bee diseases, providing an unexpected boost to pollinator conservation.

This article is part of the theme issue ‘Infectious disease ecology and evolution in a changing world’.

## 1. Introduction

Global changes such as habitat loss, anthropogenic movement of animals and plants and climate change, can impact the evolutionary ecology of infectious diseases. These perturbations may, for example, increase contact rates between species and thereby increase the risk of disease emergence [1,2], or lower the resistance or tolerance of hosts to disease through exposure to environmental stressors such as pesticides and antibiotics ([3], but see [4]). Anthropogenic changes to habitat and biodiversity, either through habitat loss or restoration efforts, can have particularly far-reaching impacts on disease ecology. Species’ abundance, biodiversity and disease are intimately linked, with disease potentially driving population declines, the loss of biodiversity and shaping community composition [5]. At the same time, the composition of host communities can play an important role in disease transmission dynamics, potentially increasing or decreasing transmission. Communities of pollinating insects are a case in point: pollinating insects and their pathogens exist in complex multi-host-multi-pathogen communities, sharing many pathogens including important viruses [6,7]. Several pathogens and parasites have switched to new host species in this system over the last century [8], exposing novel communities to diseases and leading to epidemics. Interspecific transmission in pollinators is facilitated by the sharing of floral resources, which can serve as an important hub of intra- and interspecific disease transmission for orally transmitted pathogens [9–11].

Wild and managed pollinator species, which are essential both for maintaining food security and biodiversity by pollinating crops and wildflowers [8], have experienced declines and extinctions driven by the interacting anthropogenic pressures of habitat loss, environmental stressors and emerging diseases [12]. Global anthropogenic change has further increased the risk of disease emergence in this system [6]. Agricultural intensification in Europe following World War II has resulted in the widespread loss of semi-natural habitats such as hedgerows [13]. In the UK for example, nectar resource abundance and diversity steeply decreased in the last century [14,15], with concurring declines in pollinator abundance and diversity [16]. Agri-environment schemes (AES), dating from the mid-1980s [17], were set up to encourage landowners to counteract such losses in Europe [18]. The most prominent measure targeting pollinating insects is the planting of wildflower strips to enhance the provision of nectar and pollen alongside cropped fields. Such schemes have often been successful at increasing the population size and diversity of the targeted species [19,20], which are mainly widespread social bees, while being less successful for rare species [16,21]. In addition to such local schemes, the quality of the surrounding landscape also affects pollinator diversity and abundance; for example, land cover diversity positively affects insect pollinator diversity [22] and the response of pollinators to conservation schemes can be moderated by landscape context and farmland type [23].

While the provision of wildflower strips as hotspots of floral resources can increase local pollinator abundance and diversity, it may also alter pollinator behaviours and interactions [24], thereby influencing multi-host disease transmission dynamics. If the addition of patches of floral resources in an otherwise barren agricultural landscape results in higher indirect contact rates within and between species via flower visits, we would expect higher prevalence rates for density-dependent pathogens where host biodiversity and abundance is high, as potentially indicated by studies in urban and rural areas [25–27]. Indeed, increased bumblebee density, and thus visitation rate, has been experimentally shown to increase transmission and prevalence for slow bee paralysis virus, but not the highly transmissible trypanosome *Crithidia bombi* [28]. If resources such as flower strips increase contact rates, they could thus serve as transmission hubs. However, if host species vary in their competency and susceptibility, and thus their transmission potential, then a more biodiverse host community will reduce successful disease transmission events [29]. This ‘dilution effect’ hypothesis (reviewed in [30]) predicts that biodiversity is protective against disease risk. Recent field studies have found patterns that are at least partly consistent with a dilution effect in pollinator communities [31–34], which may be lost with declines in bee diversity. If restoration measures such as adding wildflower strips increase biodiversity, we may thus expect a dilution effect depending on the variation in competency and susceptibility of the host species. Some host species can also disproportionately affect disease risk, thus the addition or increase of such species, i.e. a change in species composition, rather than altering biodiversity *per se*, can affect disease risk [35]. Commercial pollinators, particularly *Apis mellifera*, but also commercial bumblebees, may act as species-level ‘superspreaders’ of disease [36–39], similar to the effect of host transmission heterogeneity in West Nile disease [40]. If restoration measures increase the abundance of such superspreaders, transmission may increase for the relevant pathogens.

Understanding how conservation and restoration measures such as planting wildflower strips affect disease transmission, prevalence and pathogen load is key to the mitigation of disease and conservation of wild bees. To understand these complex interactions, we recorded plant and pollinator diversity and their networks on conventional farms in the UK that implemented AES for pollinators under the Higher Level Stewardship scheme (HLS farms) as well as farms that did not participate in pollinator conservation schemes but were part of the widely spread Entry Level Stewardship scheme (ELS farms). We screened over 5000 pollinating insects (including social bees, other wild bees and flies) for RNA viruses (DWV-A and -B, as well as ABPV) with different host spectra and recent epidemiological histories, based on community-level RNASeq profiles (V. Doublet *et al*. 2022, unpublished data). Acute bee paralysis virus (ABPV) is an established multi-host pathogen common both in honeybees and wild bumblebees [41], whereas deformed wing virus (DWV) is predominantly a honeybee virus that spills over into wild bumblebees [37,38] as well as other insects, and is associated with elevated honeybee colony mortality [42]. Following the anthropogenic acquisition and spread of the ectoparasitic *Varroa* mite, a viral vector that spreads DWV in honeybees, DWV-A and, more recently, DWV-B, are emerging as rapidly expanding epidemics [37,43,44]. Plant, pollinator and pathogen community composition and their network of interactions will all vary across a season, changing indirect contact rates via shared floral resources within and between insect species and will thereby potentially affect disease transmission dynamics. We, therefore, followed these communities across an entire year to explore whether HLS pollinator schemes affect disease prevalence and load across different host species and viral pathogens with different epidemiologies, potentially showing dilution or amplification of transmission in relation to the restoration measure of establishing wildflower strips for pollinator conservation.

## 2. Material and methods

### (a) Site selection

Sampling took place at 10 farms across central/southern England: five of these farms were participating in the Higher Level Stewardship (HLS, http://publications.naturalengland.org.uk/publication/2827091) agri-environment scheme (AES) for pollinators and the other five farms were either part of an Entry Level Stewardship scheme (ELS, http://publications.naturalengland.org.uk/publication/2798159) or, in the case of one farm, not part of any scheme. HLS farm management includes the delivery of selected actions to benefit the environment, such as the promotion of species diversity, the restoration of wildlife populations and the maintenance of natural resources. Here, we selected HLS farms specifically providing wildflower strips for pollinators along field margins as part of their management. All farms were at least 10 km apart, beyond the typical maximum foraging distance of honeybee workers [45] (electronic supplementary material, figure S1). We visited each farm at four time points: end of April/early May, June and August 2016 and March/April 2017.

### (b) Flower diversity

Flower diversity and plant-pollinator networks were recorded along transects for each site and time point. The number (two or three), precise location and length of transects (mean = 94 m, range 7–287 m) depended on flower availability at each site and time point (electronic supplementary material, table S1). We recorded the number of flowering units of flowering species in a 0.25 m^2^ quadrat haphazardly thrown every 10 m along transects. From these data, we computed Shannon's H’ diversity [46] for flowering plants across quadrats per site/time point.

### (c) Plant–pollinator networks

We recorded all insect visits to flowers by walking along the entire transects for 15 min. Transects were only performed in favourable conditions, including wind at a maximum of 5 on the Beaufort scale and a minimum temperature of 15°C in summer and 9°C in spring in the shade. Honeybees and bumblebees were identified to species, with the exceptions of the species complexes *Bombus terrestris/lucorum* and *Bombus hortorum/ruderatus*, neither of which have workers that are identifiable on the wing. Other bees were identified to the genus or family level when identification was not possible in the field. Flies were classified into morpho-groups, and other less common insect visitors were identified to order. We calculated Shannon's H’ diversity of all insect pollinators (note that this includes all insect plant visitors such as robber flies and pollen beetles), allowing us to ask whether there is a general dilution effect.

### (d) Network indices

To analyse whether contact rates between competent hosts are affected by land management, we used the bipartite package v. 2.1.6 [47] in R [48] to calculate pollinator–plant network indices separately for the DWV and ABPV datasets, restricting the pollinators to species that we observed to contribute to transmission, i.e. where at least one individual tested positive by virus-specific PCR as described below. For DWV-A and -B the network dataset was the same, and included *A. mellifera, Andrena* sp., *Anthophora plumipes, Lasioglossum* spp., *B. lapidarius*, *B. terrestris/lucorum*, *B. hortorum/ruderatus*, *B. pascuorum*, *Scathophaga stercoraria* and *Syrphidae* spp. (note, while *Empididae* spp. tested positive for DWV-A and *Nomada* spp. tested positive for both DWV-A and -B, these species were not recorded during the network survey). For ABPV, *A. mellifera*, *Andrena* sp., *B. lapidarius*, *B. terrestris/lucorum*, *B. hortorum/ruderatus* and *B. pascuorum* were included*.* Network indices were calculated in early and late summer only (time points 2 and 3), as in spring (time points 1 and 4) there were insufficient numbers of visited plant species to generate reliable indices. Network indices for competent hosts included 60.9% and 52.8% of all observed insect visits for DWV-A/B and ABPV, respectively (see electronic supplementary material, table S2); the majority of excluded visitors were predominantly pollen beetles (29.4% of all observations) and predatory robber flies (Asilidae, 6.2% of all observations).

To test the effect of network structure and the level of shared resources on disease prevalence and loads, we chose indices that reflect the variation in host contact networks across time and field sites. Using the *networklevel* function in bipartite, we measured network connectance—a marker of network complexity obtained from the proportion of realised connections between nodes (i.e. plant and pollinator taxa) within the network; and niche overlap for the insect host species, a measure of flower resource sharing between pollinators, calculated as the mean similarity in interaction patterns with flower species. Using the *specieslevel* function in bipartite, we measured closeness centrality of *A. mellifera* (the dominant species for DWV infection) and *B. lapidarius* (the dominant bumblebee species for ABPV infection), as the shortest distance of focal species to all other nodes in the network [47]. Both niche overlap and closeness centrality are weighted indices, thus taking into account the density of observations of plant–pollinator interactions. To control for network size, we standardised niche overlap and closeness centrality against 1000 random networks based on the null model, vaznull [49], within the bipartite R package before analysis. We computed z-scores for each observed network from the mean and standard deviation of the null models, which we used in subsequent analysis. Note, we used the raw connectance value, as vaznull constrains connectance, so we could not standardise this index using null models. Additionally, in early summer we did not record any *A. mellifera* from two sites during the plant–pollinator observations, although we know they were present as they were collected for virus detection from both sites. Thus, we calculated a z-score for *A. mellifera* closeness centrality to reflect their presence in low numbers, using an observed value of 1 and an average of the mean and standard deviation of the null models. We calculated the proportional abundance of the key hosts (*A. mellifera* and *Bombus* species, including *B*.* terrestris/lucorum*, *B. lapidarius*, *B. hortorum/ruderatus* and *B. pascuorum*) per site/time point for each virus dataset.

### (e) Landscape scale indices

The Land Cover Map (2015) [50] and UKCEH Land Cover plus: crops 2016&2017 dataset from UK Centre for Ecology & Hydrology were used to extract the area of polygons representing different habitat types in QGIS v. 3.10.10; to avoid artefacts, only polygons greater than 5 m^2^ were used. To estimate the quality of the surrounding landscape of each farm for pollinators, habitats were assessed within a 2 km radius of the centroid of transects on a farm. We assigned habitat types a label of ‘good’ or ‘bad’ for pollinators based on their nectar value [14]; crops, e.g. oilseed rape and field bean, were only assigned a ‘good’ value during their flowering season; areas classified as ‘suburban’ were assigned a ‘good’ value whereas ‘urban’ areas, which detailed inspection of satellite images revealed to be built-up sites such as recycling facilities or water treatment plants in these rural landscapes, were considered ‘bad’ (electronic supplementary material, table S3). Proportion of good land was calculated as the total area of ‘good’ habitat divided by the total area.

### (f) Virus detection—prevalence and viral load

Approximately 30 of the five most common insect pollinators were collected for virus analysis along and around the transects mentioned above; the most common species differed across sites (electronic supplementary material, table S4). We differentiated between *B. terrestris/lucorum* and *B. hortorum/ruderatus* via an mtDNA length polymorphism (electronic supplementary material, table S5). Prevalence and viral load were determined as in Manley *et al.* [37]. Briefly, RNA was extracted from laterally bisected individuals using a Trizol©/ bromo-chloropropane extraction following homogenization (Invitrogen, Carlsbad, CA, USA). For prevalence PCR detections for ABPV, DWV-A and DWV-B, RNA was transcribed using GoScript Reverse Transcriptase and random hexamer primers and PCRs were performed using GoTaq DNA Polymerase (electronic supplementary material, table S5). To detect positive samples, 5 µl of PCR product were run on 1.5% TAE agarose gel with RedSafe DNA Stain (20 000 ×). Positive and negative controls were run on every gel. Viral load was measured via two-step qPCR. To select samples for qPCR analysis, we down-sampled the PCR-positive samples by randomly selecting for three samples per site/time point (or less if there were not enough individuals in that group) (*N* = 266 for DWV-A, *N* = 261 for DWV-B and *N* = 400 for ABPV). We used 400 ng of RNA template to transcribe cDNA and qPCR reactions were performed in duplicate for each sample on a Quantstudio 6 Flex Real-Time PCR system using GoTaq qPCR Master mix for dye-based detection (Promega, electronic supplementary material, table S6). We ran two no-template negative samples per plate and carried out absolute quantification using duplicate eight-point standard curves of plasmid DNA (method S1) in a 1:10 serial dilution on each plate. Mean efficiency across plates for DWV-A was 99.8% (six plates with range of 96.98–101.60%), 94.40% for DWV-B (seven plates ranging from 91.19–96.62%) and 95.8% (13 plates ranging from 90.8–105.7%), with R2 > 0.98 across assays. DWV-A standard curve detection ranged from 42 400 000 (approx. 10.5 CT) to 4 particles (approx. 34 CT), DWV-B standard curve detection ranged from 5 050 000 (approx. 14 CT) to 50 particles (approx. 32 CT) and ABPV standard curve ranged from 7 170 000 (approx. 14 CT) to 7 particles (approx. 33 CT).

## 3. Statistical analysis

Analyses were carried out in Rstudio (v. 2021.09.1) using R (v. 4.0.0) [48]. We calculated true prevalence (with 95% confidence intervals) using the R package epiR v0.9-82 [51] and the function epi.prev, to account for assay efficiency and sensitivity, which was conservatively set at 95% [52]. We tested pairwise independence of the prevalence of ABPV, DWV-A and DWV-B using Chi-squared tests of independence and Bonferroni corrected P-values. We ran generalized linear mixed models (GLMMs) using the lme4 package v. 1.1-27.1 [53] to analyse how species, time and agri-environment scheme affected prevalence and viral load of each virus. ABPV, DWV-A and DWV-B prevalence were the response variables in separate models, with binomial error distribution and logit link function. Full models included three-way interactions between the fixed effects of species (a factor with five levels: *A. mellifera*, *B. hortorum*, *B. lapidarius*, *B. pascourum* and *B. terrestris*), time (a factor with three levels: spring, early summer and late summer 2016) and agri-environment scheme (a factor with two levels: ELS and HLS). Site was included as a random effect. For this analysis, we removed species that were not present at all three time points in both agri-environment schemes (*Lasioglossum* sp., *Empididae*, *Andrena* sp., *Nomada*sp., *A. plumipes*, *S. stercoraria and Syrphidae*). Spring 2017 was excluded from these models to allow the testing of time over a year, without replicate time points. We identified the minimum adequate model via model comparison using ANOVA, and the removal of non-significant variables. We plotted models using the sjPlot package v. 2.8.12 in R [54]. Following the method above, we ran similar models with viral load of each virus as the response variable, using GLMMs with gamma error distribution and log link function. Viral loads were log transformed prior to analysis.

We used structural equation models (SEM; piecewiseSEM v. 2.1.2, [55]) to examine indirect effects of agri-environment scheme and proportion of good land on viral prevalence or load. We separately asked whether indirect effects on disease ecology were mediated via effects on *pollinator diversity* and on *plant–pollinator networks*. The first question asks whether there is a general dilution effect, i.e. does increasing diversity of potential hosts, which may differ in their capacity to transmit a certain virus, lead to a decrease in virus prevalence and load? The second analysis specifically asks whether contact rates between competent hosts are affected by these landscape factors, and subsequently affect disease ecology. For the latter analysis, we therefore restricted the infection and network data to species that were PCR-positive for the respective pathogen. For DWV-A and -B, the tested network indices were connectance, *A. mellifera* centrality, pollinator niche overlap and proportion of *A. mellifera*. For ABPV models, *Bombus* proportion and *B. lapidarius* centrality replaced the *A. mellifera* variables.

We tested the effects of agri-environment scheme and proportion of good land on species diversity or network indices using linear models based on values per site (*N* = 10), and the subsequent effect of diversity or network indices on virus prevalence (using GLMMs with binomial error distribution and logit function) and load (using GLMMs with gamma error distribution and log link). Site and species were included as random effects. Because of the strong time effects on virus prevalence and viral load, we ran separate models for early summer and late summer. We checked variance inflation factors for the individual models within the SEM using the car package (v. 3.0-12, [56]) and the function vif to ensure there were no confounding variables, removing variables if vif > 5. Note, the niche overlap z-score for DWV-A and -B at one site in late summer was an outlier (−26.43) and this site was thus removed from the models; we additionally ran the models while retaining these data to check the robustness of the model (electronic supplementary material, table S14).

We ran several GLMMS and SEMs exploring the effects of agri-environment scheme as well as biodiversity and network characteristics respectively with the prevalence and viral load of the three viruses as the response variables. We, therefore, carried out a false discovery rate correction using the function p.adjust (R stats package v. 4.1.2); to allow exploration of these complex interactions, we report observations as tentative in the discussion if false discovery rate correction did not confirm significance at the 5% level.

### (a) Sequencing

To understand the role of host communities and other ecological factors on disease prevalence, it is important to consider whether these viruses are freely transmitted within their host communities, or whether transmission events are strongly host specific, as well as whether infections are spreading or contracting over the recent past. To explore the host specificity and demographic history of the studied viruses, we analysed their sequences. For ABPV we chose all samples that showed a medium to strong band on gel electrophoresis post PCR (*N* = 129). For DWV-A and DWV-B, we divided individuals showing strong bands into *Apis* and non-*Apis*, and randomly down-sampled *Apis* to approximately 100 individuals (across site/species for each virus) and non-*Apis* to three individuals per virus/site/species (DWV-A *Apis N* = 102, non-*Apis N* = 122; DWV-B *Apis* = 106, non-*Apis* = 93). We designed virus-specific primers (electronic supplementary material, table S7): for ABPV we amplified two fragments, and for DWV-A and DWV-B we amplified three fragments of the genome by PCR as described above. For DWV-A, there was poor amplification across all three fragments in non-*Apis* individuals, thus, we sequenced a short fragment in the Lp-region of the genome for these samples, as well as the *Apis* samples for comparison (electronic supplementary material, table S8). PCR products were purified and sequenced using Sanger technology by Eurofins Genomics, Germany. Not all fragments from all samples were amplified successfully. We created alignments using Geneious (v. 10.1) by mapping the sequences to reference sequences (DWV-A: NC_004830, DWV-B: NC_006494 and ABPV: AF486072.2). We visually examined all sequence data in Geneious (v. 10.0) and only included high-quality (< three ambiguous base pairs), non-heterozygous sequences of a fragment-specific minimum length in further sequence analyses. We checked for recombination using RDP4 [57], using all implemented methods, and excluded sequences detected as recombinant (ABPV *N* = 0, DWV-A_lp−fragment_ *N* = 0, DWV-B *N* = 3). This resulted in a total fragment of 1530 bp from 87 samples for ABPV, from ORF1 with a length of 768 bp and 762 bp for the two individual fragments, respectively (OM837487–OM837660). For DWV-A, a single fragment of the lp-gene (317 bp) was successfully amplified in 81 samples from diverse host species (see electronic supplementary material, table S8 OM729321–OM729401). For DWV-B, we obtained an alignment with 86 samples, spanning 2466 bp, consisting of three fragments of the partial *helicase* (OM729488–OM72957) and *rdrp* genes (OM729402–OM72948) as well as a partial unassigned fragment of the DWV-B polyprotein (OM837401–OM837486), with individual lengths of 889 bp, 773 bp and 804 bp respectively. Thus, each virus had a distinct dataset of samples that successfully amplified across all fragments. To estimate ABPV's evolutionary rate, we used an archival collection of bumblebees collected in Switzerland between 2001 and 2011 to amplify two fragments of 875 bp from 157 individuals from the *rdrp* gene and 560 bp from the *vp1* -gene from 117 individuals (OM885451–OM885724); see Mordecai *et al*. [58] for a thorough description of the samples and the supplementary material for detailed methods (method S3).

### (b) Population genetic and phylogenetic analyses

Detailed methods for all analyses are provided in the supplementary material (method S2). Briefly, we used DNASP v. 5.10.1 [59] to test for an excess of rare polymorphisms (Tajima's D) and to assess the degree of population structure between either collection sites or between host species (*K*_ST_ [60] and *S*_NN_ [61]), as well as the nucleotide diversity π for collection sites and host species. We only included samples with at least three representatives of a particular species or geographic site. For all alignments, we constructed phylogenetic trees in MrBayes 3.2.6 [62], using gamma-distributed site-specific general time-reversible models. All trees were plotted using Figtree v. 1.4.4 (http://tree.bio.ed.ac.uk/software/figtree/). We additionally compared demographic models using BEAST 1.10 [63]. TrN + I + G was determined as the most suitable substitution model for all viruses using Jmodeltest (v. 2.1) [64]. For all viruses, exponential growth and a relaxed exponential clock were selected based on the path sampling maximum-likelihood estimator (see supplementary material for priors). We produced Maximum Clade Credibility (MCC) trees (TreeAnnotator v. 1.10.4) to reconstruct phylogenies.

## 4. Results

**Figure 1.**
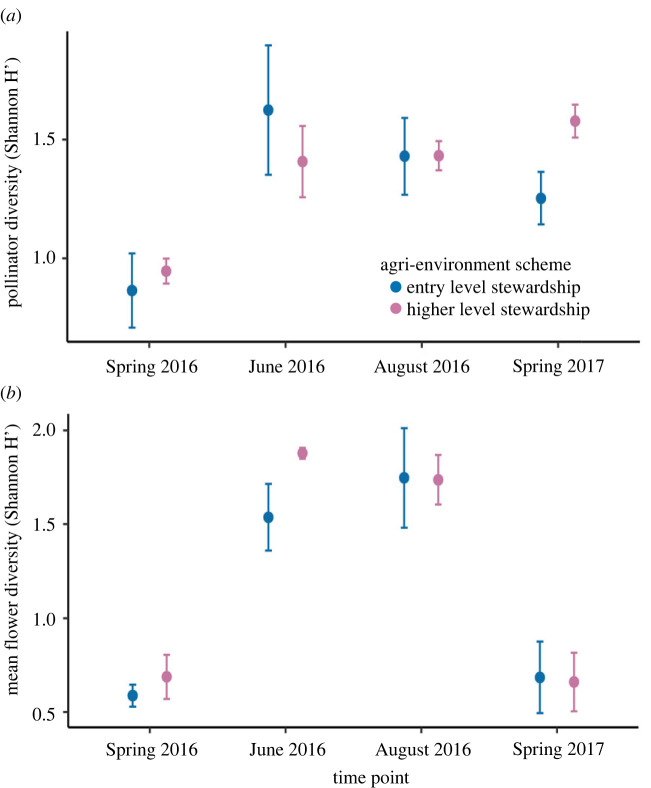
Pollinator and flower diversity. Mean (with standard error bars) pollinator (*a*) and flower (*b*) diversity (Shannon H’) by time point and scheme. (Online version in colour.)

**Figure 2.**
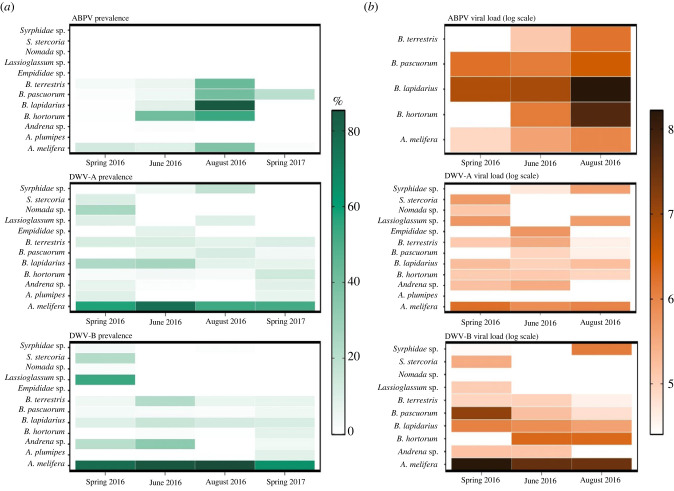
Virus prevalence and load. Mean percentage raw prevalence in a population (*a*) and mean log viral load in individual hosts (*b*) of three viruses, ABPV, DWV-A and DWV-B, across species and time. Note that viral load was only measured in spring, early and late summer 2016, and not in spring 2017, and only in species identified as positive for a particular virus at these time points. (Online version in colour.)

We screened 5180 individual pollinating insects across 12 species/genera (social bees, solitary bees and flies) for three viruses ABPV, DWV-A and DWV-B (electronic supplementary material, table S3). We collected the insects from 10 farms in south England at four time points throughout a year; spring, early and late summer 2016 and spring 2017. Across all sites and time points 62.6% (confidence intervals (CI) 61.1–64.1) of all insects were positive for one or more of the three viruses. There was species turnover throughout the year, changing species composition (electronic supplementary material, figure S2) and diversity (figure 1*a*). The effect of agri-environment scheme on pollinators and flowers was time dependent, with HLS sites supporting a higher diversity of pollinators in spring 2017 (figure 1*a*), and a higher diversity of flowers in early summer 2016 (figure 1*b*), but no difference between types of management schemes at other time points. The epidemiology of ABPV and DWV variants was strikingly different, and prevalence and viral load varied across species and time (figure 2*a,b*), while chi-squared tests of independence showed that, overall, the prevalences of ABPV, DWV-A and DWV-B were not independent of each other (spring 2016 *χ*^2^ = 224.6, early summer 2016 *χ*^2^ = 95.85, late summer 2016 *χ*^2^ = 368.32, spring 2017 *χ*^2^ = 191.17; d.f. = 2 and *p* < 0.001 for all tests).

### (a) Effects on acute bee paralysis virus prevalence and titre

ABPV was restricted to *A. mellifera* and four *Bombus* species (*B. terrestris*, *B. pascuorum*, *B. hortorum*, *B. lapidarius*) and a single *Andrena* individual (out of 779) (figure 2*a*). Across all sites and time points, prevalence of ABPV was highest in *B. hortorum* (estimated true prevalence: 38.73%, 0.95 confidence intervals (CI) 33.63–44.04, *N* = 429) and *B. lapidarius* (33.72%, CI 29.45–36.78, *N* = 812) and lowest in *A. mellifera* (11.50%, CI 9.21–14.02, *N* = 1088). ABPV prevalence was significantly higher in *Bombus* compared to *A. mellifera* (test of proportions: *χ*^2^ = 58.53, d.f. = 1, *p* < 0.001). In a GLMM examining the effects of time within a year, species (social bees only) and agri-environment scheme, as well as their interactions, scheme did not affect ABPV prevalence directly (ANOVA: *χ*^2^ = 0.20, d.f. = 1, *p* = 0.70), or indirectly via an interaction with time (ANOVA: *χ*^2^ = 1.96, d.f. = 1, *p* = 0.17) or species (ANOVA: *χ*^2^ = 8.86, d.f = 4, *p* = 0.06); agri-environment scheme was thus removed from the minimal adequate model. Time-point and species directly affected ABPV prevalence, with the highest prevalence in late summer (GLMM: estimate ± s.e. = 1.48 ± 0.24, *p* < 0.0001, (corrected fdr *p*-value < 0.001), figure 2*a*, electronic supplementary material, table S9a).

Mean viral load was also significantly affected by species and time point, but agri-environment scheme did not affect ABPV viral load directly (ANOVA: *χ*^2^ = 0.47, d.f. = 1, *p* = 0.49), or through an interaction with time (ANOVA: *χ*^2^ = 0.75, d.f. = 2, *p* = 0.69) or species (ANOVA: *χ*^2^ = 2.78, d.f. = 4, *p* = 0.59), and was removed from the model. ABPV viral load was highest in late summer (figure 2*b*, gamma GLMM with log link: estimate ± s.e. = 0.31 ± 0.05, *p* < 0.0001 (corrected fdr *p*-value = 0.05), electronic supplementary material, table S8b). Viral loads ranged from 1000 to 10^12^ virus particles per whole insect. All four bumblebee species had significantly higher ABPV viral load than *A. mellifera* (electronic supplementary material, table S9b).

### (b) Effects on deformed wing virus prevalence and titre

Both DWV-A and -B had a broader host range than ABPV: in addition to *A. mellifera* and the four bumblebee species, we also found both these viruses in solitary bees (*Anthophora plumipes*, *Lasioglossum* spp., *Andrena* spp., *Nomada* spp.) and flies (*Syrphidae* (hoverflies) and *Scathophaga stercoraria* (dung flies) (figure 2*a,b*). Only DWV-A was detected in *Empididae* (dagger flies) (four positive individuals out of 40 screened). Both viruses have significantly higher prevalence in *A. mellifera* (estimated true prevalence: DWV-A 58.78% (CI 55.5–62.1), and DWV-B 81.86% (CI 79.0–84.5), *N* = 1088), compared to *Bombus* (DWV-A: 6.64% (CI 5.39–7.9) and DWV-B: 2.59% (CI 1.54–3.72), *N* = 2787) (test of proportions: DWV-A: *χ*^2^ = 937.96, d.f. = 1, *p* < 0.001; DWV-B: *χ*^2^ = 2001.5 d.f. = 1 *p* < 0.001). Of the two viruses, DWV-B prevalence was significantly higher in *A. mellifera* compared to DWV-A (test of proportions: *χ*^2^ = 107.4, d.f = 1, *p* < 0.001), and DWV-A prevalence was significantly higher in *Bombus* compared to DWV-B (test of proportions: *χ*^2^ = 22.0, d.f = 1, *p* < 0.001).

**Figure 3.**
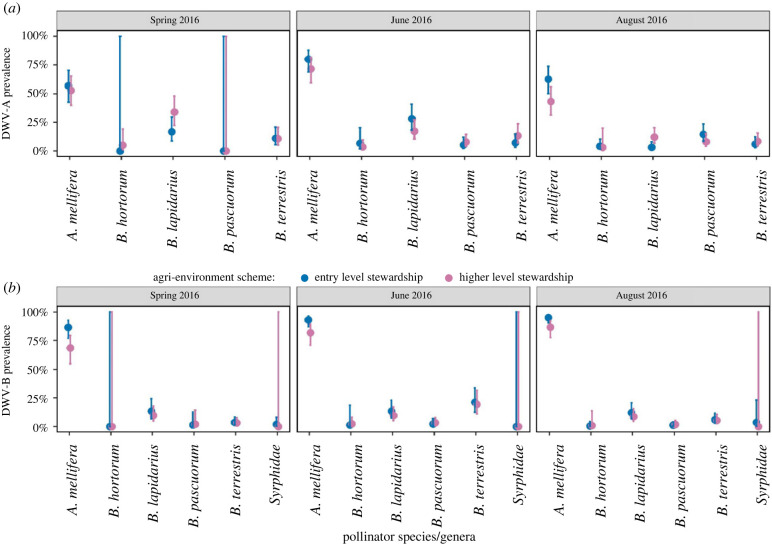
Predicted probabilities for (*a*) DWV-A and (*b*) DWV-B prevalence across species, conservation scheme and time (spring, early summer and late summer 2016; note the data from spring 2017 were excluded to allow the testing of time throughout a year without replicate time points) based on binomial GLMM models (see electronic supplementary material, tables S9 and S10 for full results). (Online version in colour.)

While agri-environment scheme did not directly affect DWV-A prevalence in a GLMM, including a three-way interaction between scheme, time point and species improved the fit of the model (ANOVA: *χ*^2^ = 22.88, d.f. = 8, *p* = 0.004). HLS management was associated with reduced DWV-A prevalence in *A. mellifera* in early and late summer, but the effect on other species is minimal (figure 3*a*, electronic supplementary material, table S10a). In a GLMM, all bumblebees were predicted to have lower DWV-A prevalence than *A. mellifera* (electronic supplementary material, table S8a). DWV-A prevalence peaked in early summer (GLMM: estimate ± s.e. = 1.11 ± 0.32, *p* < 0.001 (corrected fdr *p*-value < 0.001), electronic supplementary material, table S10a). However, the effect of time on DWV-A prevalence was species dependent, with prevalence in *A. mellifera* predicted to be four times higher in early summer than in spring, while *B. terrestris* and *B. lapidarius* were predicted to have reduced prevalence in early and late summer, respectively, compared to spring (the estimates were too uncertain in other species) (electronic supplementary material, table S10a).

The model fit for a GLMM examining the effect of species, time point and agri-environment scheme on DWV-A viral load was improved with the inclusion of an interaction between species and time point (ANOVA: *χ*^2^ = 15.21, d.f = 6, *p* = 0.02), but scheme had no impact on DWV-A viral load, and was removed from the model (ANOVA: *χ*^2^ = 0.13, d.f. = 1, *p* = 0.72). All species tested had a lower DWV-A viral load compared to *A. mellifera* (figure 2*b*, electronic supplementary material, table S10b). Viral loads varied greatly between individuals: for *A. mellifera*, the number of viral copies ranged from undetectable up to 10^12^ viral particles per bee; the majority of individuals sat around the mean of 10^5^ viral particles, with only nine individuals with loads greater than 10^6^. Despite having tested positive on PCR, 38% of individuals had an undetectable viral load via our DWV-A qPCR assay (total *N* = 119/314: by species *N* = 10 *A. mellifera*, 5 *Andrena* sp., 3 *A. plumides*, 30 *B. lapidarius*, 31 *B. terrestris*, 9 *B. hortorum*, 27 *B. pascuorum* and 2 *Syrphidae* sp.). This could be explained by the low viral loads seen for DWV-A positive samples compared to DWV-B positive samples (electronic supplementary material, figure S3) and the different methods of cDNA preparation for end point PCR versus qPCR; for qPCR, in order to compare viral quantities across samples we normalized the RNA to 400 ng before transcription to cDNA. Thus, low viral loads may have been diluted below the qPCR assay detection limit.

All species had a lower prevalence of DWV-B compared to *A. mellifera* (figure 2*a*). Time had a strong impact on DWV-B prevalence (binomial GLMM: early summer estimate ± s.e. = 0.73 ± 0.25, *p* = 0.004 (corrected fdr *p*-value = 0.03); late summer estimate ± s.e. = 1.09 ± 0.25, *p* < 0.001 (corrected fdr *p*-value < 0.001), electronic supplementary material, table S11a). The models predicted that it was twice as likely to observe DWV-B in early summer compared to spring, and three times× more likely to observe DWV-B in late summer compared to spring (electronic supplementary material, table S10a). This time effect was species dependent, with prevalence increasing in *A. mellifera* over time, but decreasing in other species with the exception of *B. terrestris* in early summer (electronic supplementary material, table S11a). Including agri-environment scheme as a direct effect significantly improved the model fit (ANOVA: *χ*^2^ = 12.42, d.f. = 4, *p* = 0.01), with a negative impact on DWV-B prevalence, predicting less prevalence of DWV-B in HLS sites compared to ELS, although this tentative effect was diminished after correction (estimate ± s.e. = −1.08 ± 0.41, *p* = 0.01 (corrected fdr *p*-value = 0.06)). As with DWV-A, this effect was driven by *A. mellifera* (figure 3*b*).

All species had a lower DWV-B viral titre than *A. mellifera* (figure 2*b*; electronic supplementary material, table S11*b*). The range of DWV-B titres in *A. mellifera* is high (10^3^–10^12^) with a mean of 10^7^, while *Bombus* titre ranged from 10^2^–10^7^, and flies and solitary bees ranged from 10^2^–10^6^. Agri-environment scheme also had a direct negative impact on titre (gamma GLMM: estimate ± s.e. = −0.10 ± 0.03, *p* < 0.001 (corrected fdr *p*-value = 0.03), electronic supplementary material, table S11b), with lower titre predicted in HLS sites (electronic supplementary material, figure S4). Titre was also lower in late summer (gamma GLMM: estimate ± s.e. = −0.10 ± 0.04, *p* < 0.03), although this tentative effect was diminished after correction (corrected fdr *p*-value = 0.13). Interactions between species and time (ANOVA: *χ*^2^ = 2.32, d.f. = 6, *p* = 0.88) and species and scheme (ANOVA: *χ*^2^ = 1.00, d.f. = 4, *p* = 0.90) did not improve the fit of the model.

### (c) Dilution or amplification effects

Using structural equation models, we tested whether agri-environment scheme and the surrounding landscape (proportion of good land for pollinators) affected pollinator species diversity (H’) in early and late summer, which in turn could affect the prevalence and viral load of each virus. We found no effect of scheme or proportion of good land on pollinator species diversity, and species diversity had no effect on ABPV prevalence or viral load (electronic supplementary material, table S12a and b). There was also no direct effect of species diversity on DWV-A prevalence or viral load. In early summer we observed a tentative reduction in DWV-B viral load (but not prevalence) in the HLS scheme (gamma GLMM: estimate ± s.e. = −0.14 ± 0.06, *p* = 0.02), although this effect was diminished after correction (corrected fdr *p*-value = 0.11; electronic supplementary material, figure S5). Similarly, DWV-B load was reduced with increasing pollinator diversity (gamma GLMM: estimate ± s.e. = −0.12 ± 0.06, *p* = 0.02), but again this tentative effect was diminished after correction (corrected fdr *p*-value = 0.11; electronic supplementary material, table S12b).

### (d) Plant–pollinator networks and transmission

**Figure 4.**
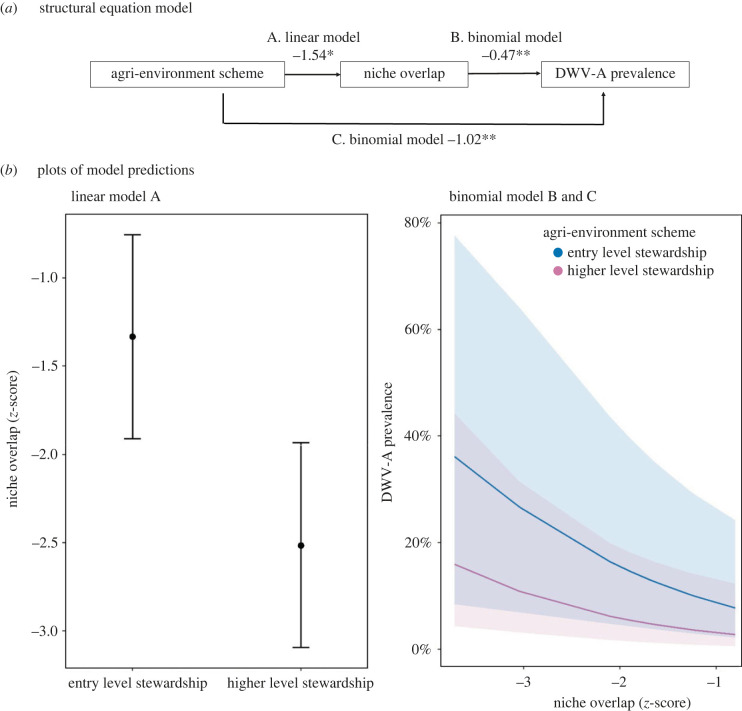
Drivers of DWV-A prevalence and viral load. (*a*) Structural equation model (SEM) showing GLMM model type, estimates and significance (*p < 0.05, **p < 0.01) of direct and indirect relationships between conservation scheme (Entry Level Stewardship and Higher Level Stewardship), interspecific pollinator niche overlap and DWV-A prevalence in early summer. Non-significant terms are not shown in the model illustration. (*a*) Linear models were based on site variables (*N* = 10), testing the effect of agri-environment scheme and proportion of good land on network indices. Binomial models with logit link (*b,c*) were run on raw prevalence data, testing the effect of network indices on DWV-A prevalence. For full model results see electronic supplementary material, table S12c. (*b*) Plots of the predictions of models A, B and C based on individual GLMMs, rather than the SEM. The predicted values of niche overlap at Entry Level Stewardship compared to Higher Level Stewardship sites for the linear model (A). The predicted proportions of DWV-A prevalence across sites with differing niche overlap values and agri-environment schemes for the binomial models (B and C). (Online version in colour.)

The proportion of *Bombus* positively affected ABPV prevalence (binomial GLMM: estimate ± s.e. = 2.88 ± 0.81, *p* < 0.001 (corrected fdr *p*-value < 0.001); electronic supplementary material, figure S6 and table S13a) in late summer, when the virus becomes more prevalent. ABPV viral load was not affected (electronic supplementary material, table S13b). For DWV-A and -B, and the broader range of species they infected, HLS management in early summer reduced the level of niche overlap between pollinator species, likely due to the provision of diverse flora at this time (figure 1*b*), although this tentative effect was diminished after correction (linear model: estimate ± s.e. = −1.54 ± 0.63, *p* = 0.04 (corrected fdr *p*-value = 0.26); figure 4*a*, electronic supplementary material, table S13c). Lower niche overlap reduces contact between species, i.e. how much insect visitors share flower species, and may potentially reduce interspecific disease transmission and thus disease prevalence and load across the community. However, to the contrary, we found a negative relationship between niche overlap and DWV-A prevalence (not load) in early summer (DWV-A prevalence: estimate ± s.e. = −0.47 ± 0.16, *p* = 0.003 (corrected fdr *p*-value = 0.03); figure 4*b*, electronic supplementary material, table S13c), which suggests that more interspecific contact between competent pollinators actually may lead to a reduction in DWV-A, i.e. a dilution effect. Because DWV transmission in honeybees is expected to be dominated by within-hive transmission via the *Varroa* mite [65], with indirect oral transmission to other species via for example flower visits [37,66], we tested whether this effect remains when we additionally ran models separately for honeybees and non-*Apis* bees. The taxa-specific models confirmed the effect (electronic supplementary material, figure S7*a,b*). Additionally, we demonstrated that niche overlap was not dependent on honeybee density (Pearson's correlation coefficient = 0.15, d.f. = 8, *p* = 0.67).

Despite this indirect positive relationship between HLS management and DWV-A via niche overlap, we found a direct negative effect of HLS management on DWV in early summer: DWV-A prevalence was predicted to be lower in HLS compared to ELS (binomial GLMM: estimate ± s.e. = −1.02 ± 0.37, *p* = 0.006 (corrected fdr *p*-value = 0.046); figure 4*b*, electronic supplementary material, table S12c); and DWV-B viral load (not prevalence) was also predicted to be lower in HLS compared to ELS (gamma GLMM: estimate ± s.e. = −0.19 ± 0.08, *p* = 0.02), although this tentative effect was diminished after correction (corrected *p*-value = 0.12; electronic supplementary material, table S12f). None of the above relationships were observed in late summer.

### (e) Viral population genetics

**Figure 5.**
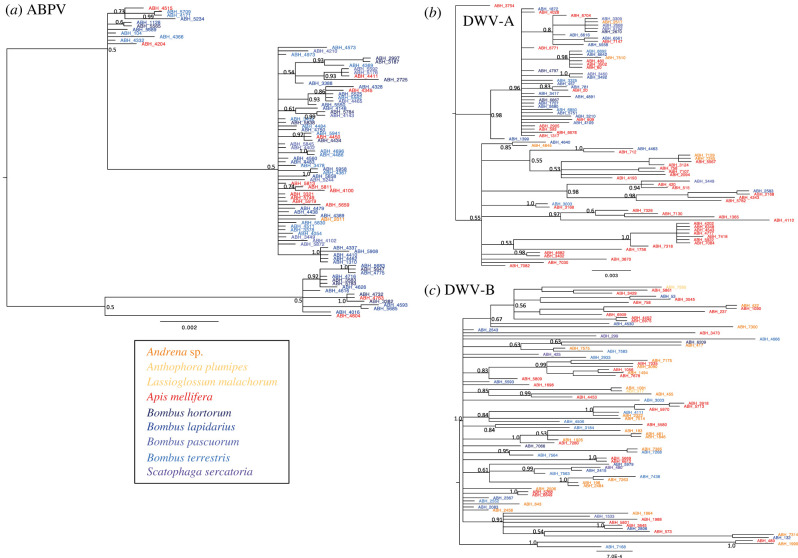
Bayesian phylogenetic trees for ABPV (*a*), DWV-A (*b*) and DWV-B (*c*). Numbers indicate posterior support up to the third node. Colours indicate host species (see legend). (Online version in colour.)

The population genetic analysis revealed that ABPV and DWV-A showed a different recent epidemiological history compared to DWV-B, as can be seen in the reconstructed phylogenies (figure 5*a–c*). DWV-B showed a strong excess of single nucleotide polymorphisms (Tajima's *D* = −2.483, *p* < 0.01 and very low genetic diversity *π* = 0.005), in contrast to DWV-A and ABPV (*π_DWV−A_* = 0.018 and *π_ABPV_* = 0.011, *p* > 0.05). For DWV-B, there was no genetic differentiation by host species and only weak differentiation by collection site (*K*_st_ = 0.031, *p* < 0.01; *S*_nn_ = 0.23, *p* = 0.039). DWV-A and ABPV on the other hand both showed some genetic differentiation by host species (ABPV: *K*_st_ = 0.053, *p* = 0.019; *S*_nn_ = 0.378, *p* < 0.001; DWV-A: *K*_st_ = 0.068, *p* < 0.001; *S*_nn_ = 0.473, *p* < 0.01) and stronger differentiation by collection site (ABPV: *K*_st_ = 0.177, *p* < 0.001; *S*_nn_ = 0.507, *p* < 0.001; DWV-A: *K*_st_ = 0.149, *p* < 0.001; *S*_nn_ = 0.368, *p* < 0.001).

Temporal reconstruction in BEAST showed that within the sampled population in southern England, ABPV had the most distant root age (1942, 95% HPD = 1867–1996), with a more recent date for DWV-A (1994, 95% HPD = 1977–2008) and the most recent root age for DWV-B (2010, 95% HPD = 2007–2013). Demographic reconstruction supported exponential growth for all three viruses. However, the rate was by far the highest for DWV-B, with an exponential growth rate of 1.138 (95% HPD = 0.538–1.83), followed by DWV-A with 0.175 (95% HPD = 0.017– 0.346) and ABPV with 0.065 (95% HPD = 0.07–0.141). This equates to population doubling rates of 0.6 years, 4 years and 10.7 years respectively for DWV-B, DWV-A and ABPV in these populations (doubling rate = ln(2)/growth rate). In combination, this showed that DWV-B is undergoing a very recent and rapid population expansion in these populations, showing very little geographical differentiation and no genetic differentiation by host species. In comparison, DWV-A and ABPV, while showing population growth, are progressively older epidemics that do not currently show rapid expansion in the sampled populations. They have accrued geographical population structure and show some degree of genetic differentiation by host population.

## 5. Discussion

We explored if pollinator conservation and restoration measures in the UK Higher Level Stewardship scheme could affect viral disease prevalence and load across species-rich pollinator communities in complex agricultural landscapes over the course of a year. In summary, we found that the interactions between plants, pollinators and pathogens vary considerably across time, highlighting both conservation potential and the need to study these interactions at multiple time points. ABPV is confirmed as an established multi-host pathogen of social bees, with prevalence being driven by the proportion of bumblebees in the host community rather than being impacted by wider pollinator diversity or the network structure. For the emerging honeybee pathogen DWV, on the other hand, we found evidence for reduced prevalence in the HLS scheme, with tentative effects of general insect diversity and niche overlap of competent hosts being consistent with a dilution effect.

As expected, higher-level stewardship has a positive effect on flower species diversity, but this effect is time-dependent: flower diversity was only higher in HLS in early summer (June), while in the spring and in late summer there was no detectable difference between HLS and ELS, highlighting that these schemes currently often fail to provide floral resources for pollinators throughout the season [19]. The type of agri-environment scheme did not generally affect pollinator diversity but did alter elements of plant–pollinator networks: pollinator niche overlap was reduced in HLS farms in early summer, presumably because of the increased floral diversity [24]. This effect was absent in late summer, when floral diversity in HLS sites was similar to those of ELS sites. Strong temporal variability across plant, insect and host communities is a striking feature of this system and highlights that seasonality needs to be taken into account both in the design and in the evaluation of such conservation schemes, as well as in testing fundamental theory in wild populations.

When analysing the dynamics of two important bee viruses, we found ABPV and DWV to show markedly different phylogenetic and epidemiological patterns. ABPV was largely restricted to social bumblebees and honeybees, with both prevalence and viral load lowest in honeybees. By contrast, both strains of DWV showed a broader host range, including many solitary bee and fly species as well as the social bees; for both DWV strains, prevalence and viral load was by far highest in honeybees. This pattern is consistent with other field studies, showing that DWV-A and DWV-B are predominantly honeybee viral strains that can also spill over into a wide range of other insect species [67,68]. Accordingly, phylogenetic analysis showed that ABPV, DWV-A and DWV-B are all multi-host pathogens. The rapidly expanding and recently emerged DWV-B showed no host genetic structure and only very limited geographical structure. DWV-A and ABPV did not exhibit rapid population expansion in the studied population, with a more distant most recent common ancestor than DWV-B, and have accrued low to moderate geographical and host population structure. However, for both DWV-A and ABPV, geographical population structure is stronger than differentiation by host. This shows that all three viruses can circulate within their local host communities, without strong barriers between different host species. Therefore, niche overlap between hosts that vary in competence for viral transmission could affect viral transmission patterns, beyond a general dilution effect.

While DWV is predominantly orally transmitted between species, vector-borne transmission has emerged in honeybees with the acquisition of the ectoparasitic mite *V. destructor* in the last century. Indeed, this additional transmission route has led to a global epidemic of DWV-A in parallel with the progress of *Varroa*'s global anthropogenic spread, shown by a high doubling rate of the virus with a most recent common ancestor for this population from South England reconstructed for 1994 (95% CI 1977–2008). This coincides with the introduction of *Varroa* to the UK, which was first reported in 1992. DWV-B on the other hand has only recently emerged and appears highly adapted to both vector-borne and direct transmission in *A. mellifera* [69], rapidly overtaking DWV-A in prevalence as also shown here, with a population doubling rate of 0.6 years as compared to 4 years for DWV-A in the studied populations. Accordingly, we find very high prevalence and titre in honeybees for both DWV strains in the present study, with very low prevalence in other insects, along with often very low titres indicating that many of these may not be competent, i.e. they are not able to transmit the virus to other individuals, particularly for DWV-A. This is consistent with experimental studies showing that these DWV strains may not readily be transmitted by species other than honeybees [70,71].

We also found that ABPV and DWV disease ecology differed markedly in how they were affected by the HLS pollinator conservation scheme, insect diversity and plant–pollinator networks. The HLS scheme was associated with a reduction in DWV-A and DWV-B, either directly or via an interaction with species and time point. This effect was driven by DWV's key host *A. mellifera* and was only present in summer. This effect may partially be explained by the increased flower diversity recorded in early summer in HLS farms, when wildflower strips are in bloom but non-sown wildflowers are scarce in ELS. This may reduce intra- and interspecific contact rates via shared floral resources in HLS sites, decreasing the potential for disease transmission. Similarly, McNeil *et al.* [72] found that bumblebees collected within low-quality landscapes exhibited the highest pathogen loads, with spring floral resources and nesting habitat availability serving as the main drivers. Increasing floral resources could also reduce nutritional stress and thereby directly increase infection resistance and tolerance in bees (e.g. [73,74]). Polyfloral diets have been shown to reduce mortality of honeybee larvae when infected with various pathogens [75,76] demonstrating the importance of plant biodiversity in resistance to infections. Additionally, several plant-specific phytochemicals also have antimicrobial activity when ingested by bees (reviewed by [77]). Honeybee foraging decisions may also play a role—honeybees may be attracted to rewarding resources such as wildflower strips [78], with DWV-positive bees showing a reduction in flight performance [79] and thus ability to reach such attractive resources, potentially resulting in a lower realised prevalence in high-quality environments.

We additionally observed potential evidence for a dilution effect for DWV. Independent of the HLS scheme, we tentatively found for DWV-A that increased niche overlap between competent hosts correlated with a decrease in DWV-A prevalence and load in early summer, even though we initially expected that transmission in honeybees would be dominated by within-hive transmission via the *Varroa* mite [37,65], with contact rates while foraging causing a potentially negligible effect. However, this result was confirmed when analysing honeybees and other bees separately. For DWV-B, we found that increased insect diversity correlated with a reduction in viral load, in line with a general dilution effect. Both strains of DWV show a large potential host range, but high variation in transmission potential, with both prevalence and viral load dramatically higher in honeybees than other insects due to the presence of *Varroa* as a viral vector of DWV in these populations [37,65]. With such skewed prevalence and transmission potential of host species, high niche overlap and an increase in biodiversity could reduce prevalence and load in honeybees by diluting transmission to competent hosts, as long as transmission is not saturated by within-hive transmission in honeybees; our estimates for true prevalence indicate that non-*Varroa* transmission can still play a significant role for DWV, with true prevalence in honeybees estimated at 58.78% and 81.86% for DWV-A and DWV-B respectively. The emergence of the *Varroa* mite as a viral vector in *A. mellifera* has clearly led to a steep increase in prevalence and load of DWV, with increased prevalence and viral loads in other insects, which themselves are not parasitized by this ectoparasite [37,65,80]. However, within populations positive for *Varroa*, the present study suggests that niche overlap can still lead to a dilution of transmission and viral loads. Above and beyond the necessary *Varroa* control, pollinator conservation schemes and high insect diversity may play a role in mitigating the prevalence and viral load of this economically important virus.

This result aligns with other studies on pollinators at more local scales. For example, Daughenbaugh *et al.* [81] showed that the probability of AnBV-1 infection in honeybees is greater in habitats with low floral diversity, and suggest that between-species transmission is modulated by local floral community. Recently, Cohen *et al.* [82] found that bee diversity reduced the parasite and pathogen richness in bumblebees in urban gardens, a dilution effect, even though the provision of resources (i.e. the size of gardens and the abundance of perennial plants) itself was associated with higher parasite and pathogen prevalence, an amplification effect.

By contrast to DWV, structural equation models taking into account biodiversity or niche overlap showed that ABPV prevalence and load were unaffected by agri-environment scheme, biodiversity or plant–pollinator network characteristics. However, an increase in the proportion of bumblebees in the community increased the prevalence of ABPV in late summer, when the prevalence of ABPV was highest. These contrasting effects can be explained by the different disease ecology of these viruses. Interactions with biodiversity depend on the nature of host–pathogen interactions. We expect stronger effects of biodiversity on disease ecology for fairly generalist multi-host pathogens [35] with heterogeneous competence: the presence of hosts with low competence can lead to the dilution of transmission [83]. For ABPV, these conditions are not met. We find a narrow host range with similar high loads in all bumblebee species examined, showing low heterogeneity in transmission potential. Here, we see no dilution effect, but prevalence being driven by the presence of the more competent hosts. While *A. mellifera* is also a competent host for ABPV [84], models suggest that within honeybee colonies, transmission by *Varroa* mites is not sustainable due to the high virulence of the virus when injected into honeybees [85]. This negative association between the mite and the virus may be illustrated here by the lower loads and prevalence of ABPV in *A. mellifera* in comparison to bumblebees, and is likely to reduce the influence of honeybees in interspecific ABPV transmission. Our results exemplify the role of host range and virulence as variables defining multi-host disease dynamics.

## 6. Conclusion

Our results suggest that restoration and conservation measures for pollinators, in addition to increasing biodiversity and abundance of insect species, can reduce the prevalence and load of key viral pathogens of pollinators, both wild and managed. However, these measures require both careful design and further monitoring. Our year-long observations showed that beneficial effects of HLS schemes were limited to certain time periods. Ensuring that nectar and pollen are provided throughout the season, with high floral diversity providing polyfloral pollen and the potential for self-medication via secondary plant metabolites should be a priority. We show that even for complex multi-host pathogen interactions in field populations, disease ecology can be affected by anthropogenic efforts to mitigate habitat loss, but that effects vary based on the nature of the host–pathogen interaction.

## Data Availability

The raw data and R-code for deriving plant– ollinator networks is available for review from the Dryad Digital Repository: https://doi.org/10.5061/dryad.msbcc2g2q [86]. The data are provided in electronic supplementary material [87].
